# Relapsing Kikuchi-Fujimoto Disease With Hemophagocytic Lymphohistiocytosis

**DOI:** 10.7759/cureus.32344

**Published:** 2022-12-09

**Authors:** Tiago Valente, Gisela B Gonçalves, Valter Duarte, Laura Baptista, Gorete Jesus

**Affiliations:** 1 Internal Medicine, Centro Hospitalar Baixo Vouga, Aveiro, PRT

**Keywords:** migratory arthralgia, urticarial rash, fever, cervical lymphadenopathy, histiocytic necrotizing lymphadenitis, hemophagocytic lymphohistiocytosis, kikuchi-fujimoto disease

## Abstract

Kikuchi-Fujimoto disease is a rare, benign, and self-limited disease of uncertain etiology, affecting mostly young female patients. It usually manifests as posterior cervical lymphadenopathy and fever. Its diagnosis is based on typical histopathological changes after the exclusion of other diseases such as lupus, lymphoma, or infectious lymphadenitis. The authors present a 47-year-old female patient with recurring episodes of high fever, urticarial rash, myalgia, arthralgia, fatigue, sore throat, and generalized lymphadenopathy. Blood tests showed increased inflammatory parameters and positive antinuclear antibodies. In the two times the patient was admitted to the hospital there were no infectious agents isolated. The patient didn’t fulfill the criteria for diagnosis of lupus or any other autoimmune disease and there was also no evidence of lymphoma or other neoplastic diseases. A positron emission tomography/computed tomography (PET/CT) was performed at the first and second hospitalizations, showing generalized lymphadenopathy. At the first hospitalization, a mediastinal lymph node biopsy was obtained, excluding lymphoproliferative or granulomatous disease. During the course of the second hospitalization, a cervical lymph node was excised for biopsy, the histopathological changes of which suggested the diagnosis of Kikuchi-Fujimoto disease. The clinical course was complicated by hemophagocytic lymphohistiocytosis, with a significant increase in inflammatory markers and liver dysfunction. The patient was treated with prednisolone 1 mg/kg/day, with complete resolution of clinical and biochemical changes.

## Introduction

Kikuchi-Fujimoto disease (KFD), also known as histiocytic necrotizing lymphadenitis, is a rare, benign, and self-limiting disease of unknown etiology and pathogenesis [1]. It occurs at young ages, mostly in people younger than 40 years old, and more often in females [1,2]. It can have an acute or subacute course, lasting from weeks to months [1]. Patients mainly present with posterior cervical lymphadenopathy (60-90% of cases) and fever [1,3]. Generalized lymphadenopathy is rare [1,2]. The lymph nodes are painful on examination [4].

Other less frequent manifestations are rash (erythematous maculopapular or urticarial lesions, generalized erythema, papules, plaques), arthralgia, fatigue, weight loss, anorexia, night sweats, myalgia, headache, upper respiratory symptoms, and sore throat [1,2,4].

The diagnosis of KFD is histological, requiring an excisional biopsy of an affected lymph node [1,4].

## Case presentation

A 47-year-old female, with a previous medical history of autoimmune thyroiditis and essential hypertension on levothyroxine and losartan, was admitted to the hospital with a 20-day history of fever, myalgia, migratory arthralgia, fatigue, anorexia, sore throat, and a pruritic urticarial rash, initially affecting the face and afterward progressing to the superior limbs, chest, and legs. The physical exam was remarkable for a generalized urticarial rash, more noticeable at the chest and limbs with palmoplantar involvement and high fever (39ºC). There were no palpable lymphadenopathies. Blood tests showed an erythrocyte sedimentation rate (ESR) of 49 mm/h, C-reactive protein (CRP) 5.10 mg/dL, lactate dehydrogenase (LDH) 346 UI/L, and ferritin 877 ng/mL. There was a positive antinuclear antibody (ANA) titer of 1/160 (nucleolar staining pattern) but with negative anti-Smith (anti-SM), anti-Sjögren's-syndrome-related antigen A (anti-SSA) and anti-Sjögren's-syndrome-related antigen B (anti-SSB) antibodies, anti-double-stranded DNA (anti-dsDNA) antibody, antineutrophil cytoplasmic antibodies, anti-citrullinated protein antibody, rheumatoid factor and with normal complement levels. Multiple potential infectious agents were excluded: negative blood and urinary cultures, SARS-CoV-2, tuberculosis, syphilis, HIV, viral hepatitis, herpes simplex 1 and 2, cytomegalovirus, Epstein-Barr virus, Parvovirus-B19, and the most common zoonosis.

The patient was submitted to a full-body contrast-enhanced computed tomography (CT), which only revealed mediastinal lymphadenopathies. A positron emission tomography (PET)/CT scan followed, revealing a more extensive metabolically active lymph node involvement not only at supradiaphragmatic but also infradiaphragmatic level, as well as two-three lymph nodes at the right posterior-lateral cervical region.

A biopsy of one of the mediastinal lymph nodes was obtained through endobronchial ultrasound, excluding lymphoproliferative, granulomatous, or an infectious process but without clarifying the diagnosis. Skin biopsies showed non-specific changes suggesting drug toxicity or a viral infection. Bone marrow biopsy and aspiration were normal.

It was decided to empirically start prednisolone at 1 mg/kg/day, tapering 10 mg of prednisolone each week until the patient was on 20 mg prednisolone, then 5 mg each week for the next two weeks, and finally 2.5 mg each week for four weeks, with positive results and complete resolution of the symptoms and laboratory findings in a month.

Five months later, the patient again developed fever, papular lesions on the face, lymphopenia, and increase in CRP and was diagnosed with SARS-CoV-2 infection, with a good response to steroid therapy.

**Figure 1:**
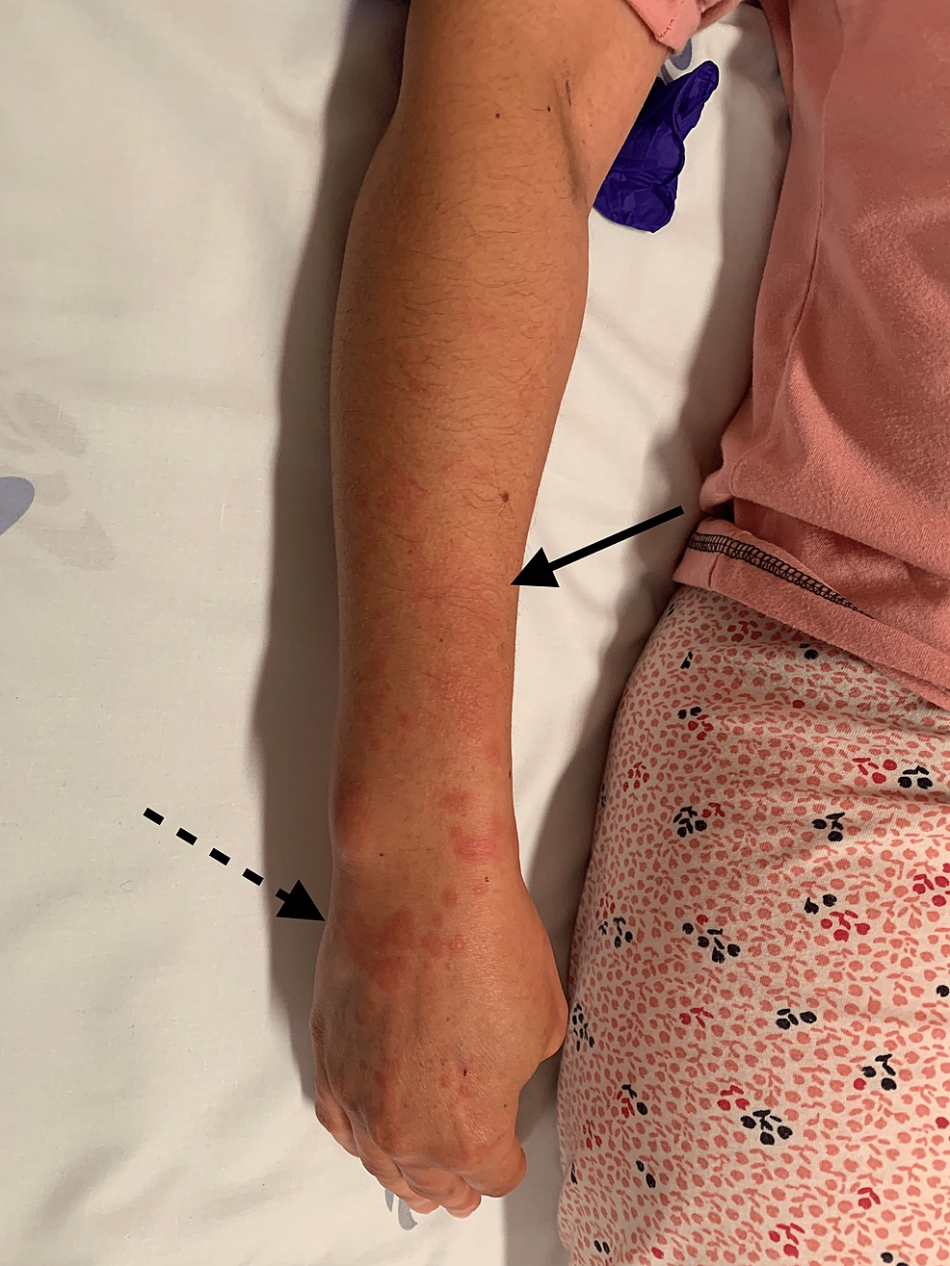
Patient’s right superior limb showing small erythematous papules coalescing to form plaques predominantly in hand and wrist (dashed arrow). In the forearm region, there is an erythematous patch (normal arrow).

**Figure 2:**
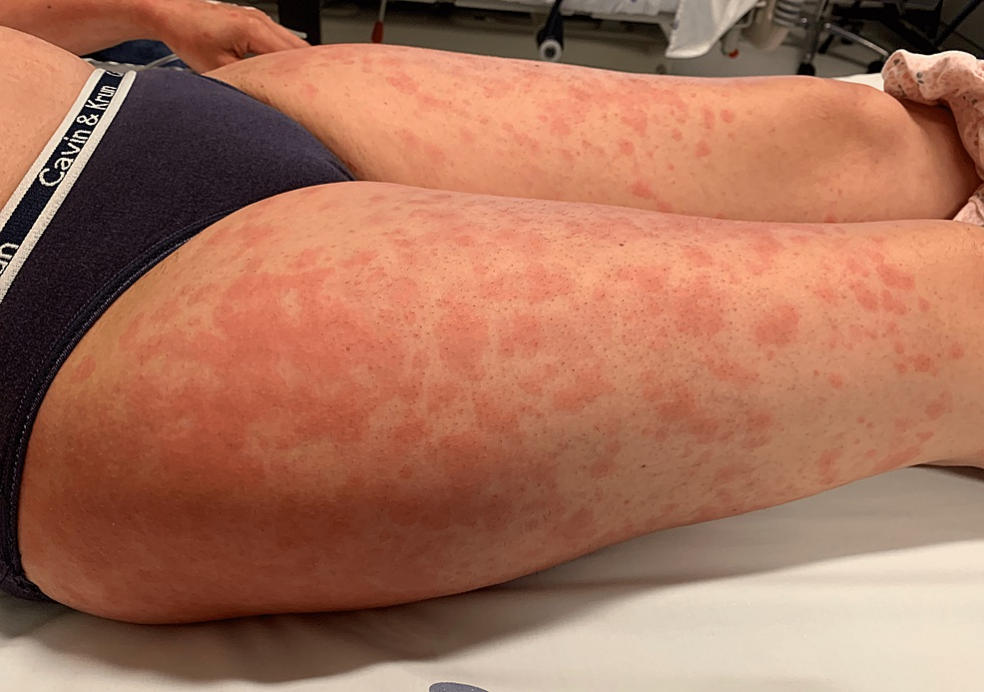
Patient’s right and left lower limbs and part of the left hand. The thighs show symmetrical, prominent erythematous plaques that had started as papules but have coalesced. Some papules and patches in the left hand are also visible.

About two months after this setback, a pruritic maculopapular/urticarial rash appeared beginning on the face, chest, and lower limbs (Figures 1 and 2). Fever ensued with a maximum temperature of 40ºC, and the patient once again complained about wrist and ankle arthralgia, fatigue, sore throat, and left neck pain, requiring readmission to the hospital. Laboratory tests showed new-onset lymphopenia (540/microL), ESR 23 mm/h, CRP 7.14 mg/dL, and ferritin 933 ng/mL. ANA titer was again 1/160 (with nucleolar staining pattern), with negative anti-SM, anti-SSA, anti-SSB, anti-dsDNA, and antiphospholipid antibodies. Multiple blood and urinary cultures were done and all were negative.

A PET/CT was once again ordered, revealing increased metabolic activity at the cervical level bilaterally and in multiple supradiaphragmatic lymph node groups, as well as infradiaphragmatic lymph node, spleen, and bone marrow involvement, the morphological evaluation also showing discreet splenomegaly.

During this hospitalization, the patient maintained significant symptoms, including high fever (39.8ºC) and general malaise, with new-onset liver dysfunction (aspartate aminotransferase 1477 U/L, alanine aminotransferase 829 U/L, LDH 4134 U/L, alkaline phosphatase 434 U/L, gamma-glutamyl transferase 968 U/L, albumin 3.1 g/dL, normal bilirubin, and no coagulopathy), hemoglobin 10.1 g/dL, mild lymphopenia, and normal platelet numbers, marked increase in CRP to 18.61 mg/dL, ESR 45 mm/h, ferritin > 16500 ng/mL, fibrinogen 394 mg/dL, and triglycerides 432 mg/dL. Abdominal ultrasound excluded both hepatomegaly and splenomegaly. This raised the hypothesis of hemophagocytic lymphohistiocytosis (HLH), with an HScore of 200 points and a probability of HLH of 80-88%. A bone marrow biopsy was performed, with no signs of hemophagocytosis or lymphoproliferative disease. A liver biopsy revealed mild inflammatory changes in resolution.

An excisional biopsy of one of the cervical lymphadenopathies was performed, which showed a massive sinusoidal infiltration by histiocytes (CD163 positive), with areas of hemorrhage and some images suggestive of hemophagocytosis, with malformed granulomas of paracortical location, delimiting areas of necrosis and a scarce lymphoid component, with a predominance of T lymphocytes (CD3 positive), predominantly in the paracortical region. The histological changes, along with the signs and symptoms presented by the patient and the exclusion of multiple other infectious, autoimmune and neoplastic diagnoses, allowed for the diagnosis of KFD.

The patient was started on dexamethasone 8 mg 12/12 hours, resulting in improvement of symptoms, decreased inflammatory markers, and decreased hepatic cytolysis and cholestasis. The patient was discharged with prednisolone 1 mg/kg/day, slowly tapering its dose during follow-up, with the resolution of symptoms, normalization of inflammatory parameters, and complete regression of liver damage.

## Discussion

The case presented by the authors has some particularities that make it rarer when compared to other cases of KFD.

First, because the manifestations of the first episode of the disease did not include the typical painful cervical lymphadenopathies (only observed on PET/CT and with low metabolic activity). Also, the patient was older when compared to the median age usually described in this disease.

Second, because it had a relapsing course, including in the context of SARS-CoV-2 infection. The recurrence rate of KFD is low (3-4%) [1]. Adults with KFD and a positive ANA title may have a higher risk of disease recurrence [3].

Third, because the patient developed HLH, a rare and potentially fatal inflammatory syndrome that, unlike the benign course of KFD, is associated with a worse prognosis and mortality [5]. A statistically significant association was observed between HLH and older age, with longer hospital stays and worse hospitalization outcomes [6]. As in this case, liver biopsy is reported to reveal reactive changes and liver enzymes revert to normal in about one month [2].

This case also illustrates the potential use and importance of PET/CT in diagnosing and predicting disease severity. The intensity of the inflammatory response in the lymph nodes and the amount of active lymph nodes detected by PET/CT may correlate with the severity of KFD [7].

This patient underwent two PET/CT scans at different times and stages of the severity of the disease. The intensity of radiolabeled glucose uptake in metabolically active lymph nodes was lower in the first (less severe) episode of the disease (mean maximum standard uptake value (SUVmax) - 4.4) when compared to the third and more severe episode (with worse clinical and analytical changes, mean SUVmax 9.2), also with the uptake of radiolabeled glucose in the spleen and spinal cord.

There is no specific treatment for KFD. Most cases resolve spontaneously or with supportive treatment (rest, analgesia, antipyretics) [1,2]. In case of a relapsing disease or a more severe clinical course, systemic corticosteroid therapy may be necessary [1]. In this case, prednisolone at a dose of 1 mg/kg/day proved to be effective in symptomatic and analytical improvement.

## Conclusions

KFD has non-specific clinical manifestations and a wide range of important differential diagnoses such as lupus, malignant lymphoma, and many infectious diseases. It should be included in the list of differential diagnoses of a young person with prolonged fever, urticarial rash, migratory arthralgia, and generalized lymphadenopathy. Missing this diagnosis might cause harm to the patient, in that he/she may be submitted to aggressive, immunosuppressive, and unnecessary treatments. Although it is mostly a benign condition, rarely it can be associated with HLH, resulting in a worse prognosis, potential multiorgan dysfunction, and death.
